# Understanding infection control needs in schools: insights from Nebraska’s school health staff

**DOI:** 10.1017/ash.2026.10396

**Published:** 2026-05-12

**Authors:** Mounica Soma, Chris Cashatt, Muhammad Salman Ashraf, Kate Tyner, Andrea Riley, Alice Sato, Robin Williams, Juan Teran-Plasencia

**Affiliations:** 1 The Nebraska Medical Center: Nebraska Medicine, USA; 2 University of Nebraska Medical Center College of Medicine, USA; 3 Nebraska Department of Health and Human Services, USA; 4 Children’s Nebraska, USA

The COVID-19 pandemic highlighted the critical importance of infection prevention and control (IPC) practices in K-12 schools. However, unlike healthcare facilities, which follow established IPC protocols and guidelines, schools often lack guidance, support, and training to implement effective IPC measures.^1-3^ In response, the Nebraska Infection Control Assessment and Promotion Program (ICAP) supported by the Nebraska Department of Health and Human Services Healthcare-Associated Infections and Antimicrobial Resistance Program, in partnership with Children’s Nebraska and the Global Center for Health Security at the University of Nebraska Medical Center, developed and disseminated IPC resources to support school health personnel. Key initiatives included a statewide IPC conference, access to high-efficiency particulate air (HEPA) filters, monthly Kids Health Zone newsletters, and the Nebraska Infection Control in Education (NICE) book, which provides guidance on state regulations, communicable disease management, and outbreak preparedness.^4,5^ We conducted a statewide learning needs assessment (LNA) to gather feedback on our school-focused resources and to better understand current knowledge gaps, training priorities, and resource preferences.

## Methods

Between September and October 2024, we distributed a REDCap LNA survey to school health personnel across Nebraska (Supplementary Appendix 1). Recruitment occurred via email to school nurse contact lists, a social media campaign, and the NE ICAP website. Participation was voluntary. The survey targeted school nurses, health paraprofessionals, and administrators in healthcare roles. Two screening questions confirmed eligibility: employment in Nebraska and work in healthcare role at a school. Eligible participants completed ten structured questions on IPC knowledge, training, and resource needs. We analyzed quantitative data using descriptive statistics, and subject matter experts thematically analyzed open-ended responses to identify key challenges and opportunities for support. We conducted additional cross-tabulation and chi-square analyses in SAS to assess associations between resource use, IPC support needs, language needs, and preferred additions to the NICE book.

## Results

Of the 88 survey responses received, 79 were eligible for analysis after nine respondents were excluded during screening questions. Respondents most frequently used the NICE book and Kids Health Zone newsletters during the past year, and Spanish was the most requested language for translated materials. The top 3 most frequently reported IPC education/support needs included outbreak management, prevention of airborne disease transmission, and environmental cleaning and disinfection practices. Respondents most frequently requested newsletter content on infectious diseases and general IPC topics. Most reported having adequate IPC supplies, though priority needs included immunization documentation, hygiene and safety supplies, and translated materials. These descriptive findings are summarized in Table 1.

**Table 1.** Findings from the school learning needs assessment survey

Survey topic (N)	Count (%)
**Resources or training opportunities utilized in the past 12 months to enhance IPC (N = 79)**	
NICE book	55 (70)
Kids Health Zone Newsletters	38 (48)
Online Infection Prevention Control in School Settings online course provided by NICN	16 (20)
Other online sources	11 (14)
NICN live conference “Infection Prevention and Control in School Settings” held on April 28th, 2023	6 (8)
None	11 (14)
**IPC topic areas that need more support or education (N = 79)**	
Outbreak management	43 (54)
Prevention of airborne diseases transmission inside facilities	35 (44)
Infection prevention measures during athletic and sporting events	34 (43)
Environmental cleaning and disinfection practices	34 (43)
Bloodborne pathogens	13 (16)
Infectious diseases	28 (35)
Immunizations	24 (30)
Bloodborne pathogens	13 (16)
Other	7 (9)
**Tools or resources of interest to be included in NICE book (N = 79)**	
Parent letters	59 (75)
Disease specific handouts	58 (73)
Posters	36 (46)
Other	8 (10)
**Specific language considerations (N = 41)**	
Spanish	32 (78)
Karen	4 (10)
Arabic	3 (7)
Other	9 (22)
**Resources that are a priority need (N = 22)**	
Immunization documentation and health regulations	7 (32)
Hygiene and safety supplies	4 (18)
Education and management of health concerns	3 (14)
Translated content	3 (14)
Parent letters	2 (9)
Educational flyers/posters/handouts	2 (9)
Professional development and training	1 (5)
**Topics of interest for the kids health zone newsletter (N = 37)**	
Infectious diseases & common illnesses	20 (54)
General IPC topics	15 (41)
Insect bites and infestations	11 (30)
Skin conditions & allergies	11 (30)
Immunization & vaccination	5 (14)
Current topics of interest	4 (11)

*Note*. IPC, infection prevention and control; NICN, Nebraska infection control network; NICE, Nebraska infection control in education.

Additional analyses identified several statistical associations. Use of the Kids Health Zone newsletter was significantly associated with lower reported needs in environmental cleaning and disinfection (71% non-readers vs 42% readers; *χ^2^* = 6.59, *P* = .01). Respondents needing outbreak-management support were significantly more likely to request disease-specific handouts (86% vs 58%; *χ^2^* = 7.71, *P* = .006). Respondents needing additional support in environmental cleaning and disinfection were significantly more likely to request disease-specific handouts in the NICE book (88% vs 62%; *χ^2^* = 6.72, *P* = .0096) and showed a trend toward greater interest in parent letters (85% vs 67%; *χ^2^* = 3.55, *P* = .059). There was no association between the number of IPC resources used and the number of IPC needs reported.

## Discussion

Schools play a critical role in preventing the spread of infectious diseases to ensure the health and safety of students, staff, and the wider community.^6^ This needs assessment identified persistent gaps in targeted IPC knowledge, multilingual resource availability, and the practical application of IPC strategies, particularly in outbreak management, airborne disease prevention, and environmental cleaning and disinfection. These findings are consistent with prior literature emphasizing the need for tailored IPC education in schools, especially in the wake of the COVID-19 pandemic. For example, Hilbert et al.^7^ found that while school staff frequently used disinfectants, many lacked training on proper use and relied on unfamiliar products. Wang et al.^8^ similarly emphasized that hygiene practices and ongoing education are foundational to disease prevention. Hass et al.^1^ demonstrated that online IPC toolkits can improve school staff confidence but still require clear, sustained guidance to support implementation. In addition, some studies^2,3^ reported that school staff had no formal training postpandemic, despite increased responsibilities, and expressed a strong need for updated policies and role clarity. Our findings further highlight the importance of pairing resource distribution with contextualized training and competency-based support,^9^ to ensure consistent and effective IPC implementation. Respondents who reported higher IPC needs were also more likely to request practical tools such as disease-specific handouts, parent letters, and translated materials. Newsletter engagement was associated with fewer environmental cleaning and disinfection needs, suggesting that targeted, accessible communication channels may help address specific gaps.

Limitations of this study include potential self-selection bias due to voluntary participation, limiting generalizability. The survey did not collect demographic variables, preventing analysis across socioeconomic, geographic, or institutional factors that may influence IPC needs and resource access. Furthermore, the assessment was conducted solely within Nebraska, and findings may not reflect IPC challenges or priorities in other states or regions with differing school health infrastructures or policies. Future studies with broader, multi-state samples should consider demographic and regional variations to enhance generalizability.

This study highlights priority IPC needs and resource gaps in schools, emphasizing the importance of standardized training, translated content, and targeted education. Future research should evaluate how staff training, continuing education, audits and feedback strategies influence IPC practices, outbreak response, and health outcomes in educational settings.

## Supporting information

10.1017/ash.2026.10396.sm001 Soma et al. supplementary material Soma et al. supplementary material

## Supplementary material

The supplementary material for this article can be found at https://doi.org/10.1017/ash.2026.10396.
