# Supplementary material for: Understanding infection control needs in schools: insights from Nebraska’s school health staff
Source: Antimicrob Steward Healthc Epidemiol. 2026 May 12;6(1):e142. doi: 10.1017/ash.2026.10396 (PMC13162065; doi:10.1017/ash.2026.10396)
Supplement: Soma et al. supplementary material [file S2732494X26103969sup001.pdf]

School Nurse Learning Needs Assessment Survey

Our programs (Nebraska ICAP, Nebraska DHHS, & Childrens Nebraska) want to better support you and your efforts to prevent infections in the school setting. Thank you for taking the time to help us assess our services and to let us know about your needs. It is anticipated to take less than 10 minutes of your time and your responses will be kept confidential. Thank you again for your participation and feedback that will be used to help plan future interventions.

Do you practice in a healthcare position in a school setting?

- ◯ Yes
- ◯ No

Do you work in a Nebraska school?

- ◯ Yes
- ◯ No

---

1. In the past 12 months, what resources or training opportunities have you utilized in to enhance your professional practice and/ or knowledge of infection prevention and control?

- ☐ NICN live conference "Infection Prevention and Control in School Settings" held on April 28th, 2023?
- ☐ Online Infection Prevention Control in School Settings online course provided by NICN (offered through Nebraska Methodist Professional Development & Continuing Education)?
- ☐ NICE (Nebraskan Infection Control in Education) book
- ☐ Kids Health Zone Newsletters
- ☐ Other on-line sources (please specify) ______
- ☐ None

---

2. What topics or areas in infection prevention and control do you feel you need more support or education on? (Choose as many as you would like)

- ☐ Prevention of airborne diseases transmission inside facilities
- ☐ Infection prevention measures during athletic and sporting events, including communal activities such as band and music.
- ☐ Environmental cleaning and disinfection practices
- ☐ Outbreak management
- ☐ Bloodborne Pathogens
- ☐ Infectious Diseases ______
- ☐ Immunizations
- ☐ Other, list as many as you wish! ______

---

3. Are there any tools or resources that you would like to see included in the NICE book?

- ☐ Parent letters
- ☐ Disease specific handouts
- ☐ Posters
- ☐ Other (please specify) ______

---

4. Are there any specific cultural or linguistic considerations that need to be addressed in the NICE book?

a. Specific language needed ______
b. Please list specific resources that are a priority need ______

---

5. Do you feel equipped with the necessary supplies and equipment to implement effective infection prevention measures?

- ◯ Yes
- ◯ No

Please add comments about what is necessary.

____

---

6. Does your school report school absenteeism weekly?

- ◯ Yes
- ◯ No

If yes, please specify method of reporting.	◯ Directly LHD<br>◯ NE DHHS REDCap<br>◯ Other (please specify) ______
If no, please specify any barriers to reporting.	__________________________________
7. For the Kids Health Zone Newsletter, please list topics of interest:	__________________________________
8. Did your school receive a free HEPA machine and filters from NE DHHS?	◯ Yes<br>◯ No

If yes, please answer the below questions:

a. How many units do you have?

______
b. How is your school utilizing the HEPA machine? ______
c. Could your school staff benefit from additional training on use and benefit of HEPA machines? ______

______ ______

d. Any questions about use of HEPA machines that were given to schools through DHHS?

______

9. Where did you hear of this survey?	☐ Facebook/Social media<br>☐ Local Health Department (LHD)<br>☐ Email from ESU<br>☐ Email from NSNA<br>☐ NSNA conference<br>☐ NE ICAP email<br>☐ Other
If Other, please specify:	__________________________
